# Reproduction in South American wild canids—A review

**DOI:** 10.3389/fvets.2022.986030

**Published:** 2022-10-24

**Authors:** Jaqueline Candido de Carvalho, Fabiana Ferreira Souza, John Patrick Kastelic, João Carlos Pinheiro Ferreira

**Affiliations:** ^1^Department of Veterinary Surgery and Animal Reproduction, School of Veterinary Medicine and Animal Science, São Paulo State University (UNESP), Botucatu, Brazil; ^2^Santo Amaro University, UNISA, São Paulo, Brazil; ^3^Department of Production Animal Health, Faculty of Veterinary Medicine (UCVM), University of Calgary, Calgary, AB, Canada

**Keywords:** biotechnology, reproductive physiology, conservation, carnivore, Canidae

## Abstract

Canids occupy the top of the food chain and are fundamental in sustaining a wild animal/environmental balance. South America, the most biodiverse continent, has 11 species of canids inhabiting diverse biomes, with or without overlapping territories. Although several species are threatened, little is known about their reproductive biology. Remarkably, basic knowledge regarding ejaculate characteristics, sexual behavior, female reproductive cycles, pregnancy and management, and parturition are scarce or absent. These gaps complicate or preclude development of conservation programs. This review compiles the current knowledge of the reproductive biology of South American canids and discusses implications of this scenario.

## Introduction

Scientific interest regarding the Class Mammalia began in the sixteenth century, motivated by livestock predation by members of the Order Carnivora, always searching for food and threatening rural populations (1). Included in the Order Carnivora, the Family Canidae, which includes 13 genera and 35 species (2), aroused great attention due to its great diversity and worldwide distribution (3).

*Despite their skulls, teeth, and muscles adapted to capture and kill prey, similar to other members of Order Carnivora, canids have evolved to acquire specific features, including more teeth and longer skulls* *(**4**), legs, and feet. Also, they usually have five toes on the forefeet and four on the hindfeet, long un-fused metapodials, no retractile claws, and digitigrade stance. In addition, all male canids have a well-developed penis bone (os penis)* *(**5**)*.

*It is noteworthy that* canids are extremely diverse and can survive under the most extreme environmental conditions, from scorching deserts to severe winters (6). Consequently, wild canids that arrived from North America, *via* the Isthmus of Panama (7), have occupied very diverse South American biomes, including the Caatinga, Atlantic Forest, Pampas, Cerrado, Amazon, Pantanal, and Chilean Desserts (8). Currently, South America, the continent with the most extraordinary canid biodiversity (2), is home to 11 wild canine species (1), with incredible potential for unique scientific discoveries (3).

Environmental challenges of South American biomes triggered a series of profound genotypic and phenotypic changes (7, 9), readily apparent by the large interspecific ecomorphological varieties in eating habits, predation behaviors, and reproductive physiology (10, 11). For example, small Bush dogs form packs, inhabit semiaquatic environments, and are carnivores (12). In contrast, the large Maned wolf, with long legs and large ears, is omnivorous and solitary (13).

Wild canids have great ecological importance in South America. Besides being sentinel species for emerging canine diseases (14), they are active participants in maintaining and balancing South American ecosystems (15), including maintenance of flora. For example, while consuming a specific fruit from the lobeira tree (S*olanum lycocarpum*), a Maned wolf (*Chrysocyon brachyurus*) acts as a seed disperser (16). Similarly, the Crab-eating fox (*Cerdocyon thous*) is a secondary disperser of *Eugenia umbelliflora* seeds (17).

Despite their importance, wild canids are seriously threatened due to loss, degradation, and fragmentation of their habitats, forest fires, hunting, and being struck by motor vehicles. Consequently, they are subject to geographic isolation, inbreeding, loss of genetic variability, decreased reproductive efficiency, and reduced population numbers (18, 19). Inbreeding is a critical cause of the increased proportion of morphologically defective sperm in ejaculates (20–25) and increased incidence of congenital reproductive conditions (e.g., monorchidism, anorchidism, cryptorchidism, and testicular hypoplasia) (21). Additionally, geographic isolation can hybridize phylogenetically similar species, such as Culpeo (*Lycalopex culpaeus*), Darwin's fox (*Lycalopex fulvipes*), and Chilla (*Lycalopex griseus*) (26, 27), generating further reproductive impacts.

A possible strategy to mitigate the loss of genetic variability and increase the wild canid population in fragile ecological niches would be to develop and optimize reproductive biotechnologies aimed at establishing germplasm and developing assisted reproductive programs in vulnerable populations at-risk, or seriously threatened species (28). However, despite establishment of the International Convention on Biological Diversity (CBD) during the UN Rio-92 meeting (29), little has been done in South America for conservation of its fauna (30). Specifically, for canids, there are virtually no consistent programs for their preservation, mainly due to no or limited financial support from funding agencies (31).

With this review, our objective is to characterize the state-of-the-art knowledge regarding reproductive biology of the wild South American canids and how this scenario influences development of reproductive biotechnologies.

## General features

Like other members of the Mammalia Class, canids are homeothermic, sexually reproducing species, with internal fertilization and intrauterine embryonic/fetal development. After birth, individuals feed predominantly on milk produced by the maternal mammary glands and maintain a close relationship with their mothers until puberty (32).

All South American canids belong to a subfamily Canidae (4). Of them, six occur predominantly in Brazil: Crab-eating fox (*C. thous*), Maned wolf (*C. brachyurus*), Hoary fox (*Lycalopex vetulus*), Pampas fox (*Lycalopex gymnocercus*), Short-eared dog (*Atelocynus microtis*), Bush dog (*Speothos venaticus*) and five in other countries, except Brazil, Culpeo (*L. culpaeus*), Darwin's fox (*L. fulvipes*), Chilla (*L. griseus*), Sechuran fox (*Lycalopex sechurae*), and Gray fox (*Urocyon cinereoargenteus*) (33).

Due to its wide geographic adaptability, the Crab-eating fox is present in most of South America (4, 34, 35), and lives as far north as Panama, in Central America (36). This great adaptability is mainly attributed to their eating habits, including insects, rodents, reptiles, birds, river crabs, eggs, and fruits (34).

The Crab eating-fox, the only species of the genus C*erdocyon*, is medium-sized and has a predominantly gray and light brown brindle coat, except on the tips of the ears, back of the legs, and between the jaws, where it is black (35). There is a longitudinal strip of darker hair along its dorsal line, and in the chest and belly, the coat is lighter (4). Adults have a body 60–70 cm long and a characteristic voluminous tail of ~30 cm. Members of this species live and hunt in pairs or extended family groups formed by parents and three juvenile animals, with larger groups being rare (11, 37).

The Maned wolf, the only species of the genus *Brachyurus*, is widely distributed in South America (38). The species has been progressively adapting to agricultural regions, due to high rodent densities. It is also present in areas of previously dense forests transformed into pastures, if there are residual niches of original vegetation (39–41). The Maned wolf, one of the most threatened South American canids (42), is considered the flagship species for preserving the Cerrado, with many efforts devoted to its preservation (43).

The Maned wolf is the largest wild canid in South America. It has a body and tail that are 94–115 and 38–50 cm long, respectively, reddish-brown ruffled fur, characteristic mane, singular ears with well-developed bursae, and a narrow skull, similar to coyotes (*Canis latrans*) (39, 44). The slender body, long limbs, and paced gait are evolutionary evidence of its adaptation to savannas (39). Since 1985, the Maned wolf has been in the Species Survival Plan (SSP) of the Association of Zoos and Aquariums (AZA), with publication of manuals describing development of conservation strategies (45, 46).

The Short-eared dog, the only species of the genus *Atelocynus* (4), is medium-sized (47) and considered the only endemic canid of the Amazon region, where it inhabits low-anthropic-disturbed plains near the margins of the rivers (4), living in burrows or hollow tree trunks together with the young (47). They are solitary animals. They tolerate living in pairs in captivity but do not have social interactions with their peers (4). Due to its low population density, it is rarely seen, and it is the least studied wild canid in the world. Consequently, its geographical distribution is poorly documented (48), and little is known about its habits, ecology, reproduction, or behavior (47, 49). The Short-eared dog differs from other South American canids in having an elongated head, small ears, dense fur (4), and partial interdigital membrane, compatible with its aquatic habitats (50). Its coat is dark gray or reddish, mixed with white hairs, including the long and hairy tail (50). It has a varied diet, predominantly fruits, insects, small and medium-sized mammals, fish, crabs, amphibians, and carrion (47).

The Bush dog, the only species of the genus *Speothos*, is rarely observed in the wild despite having diurnal habits. It is present in several Central and South American regions, including southern Panama, Guyana, Suriname, Venezuela, Colombia, eastern Peru, eastern Bolivia, Paraguay, and northeast Argentina. In Brazil, it occurs in the Amazon, Cerrado, Atlantic Forest, and Pantanal (51). Despite being short, the size of its members exceeds those of the animals of the genus *Aletocynus*, which is probably an evolutionary adaptation to its habitat of humid forests, gallery forests, and places close to watercourses. Unlike other neotropical canids, the Bush dog has a broad head, elongated body, short snout, short limbs and tail, and small, rounded ears (52). It also has a characteristic footprint with five pads, due to the first palm digit imprint (53). Adults have a reddish coat, which can vary from darker to yellowish tones, whereas puppies are grayish (52). They are gregarious animals that live and hunt in family groups of up to 10 individuals (54). They have a strictly carnivorous diet and, due to their group organization, can prey on medium and large animals, including pacas (*Cuniculus paca*), coatis (*Nasua nasua*), and even small deer (*Mazama* spp.) (52). They have a vast vocal repertoire, with an ability to mimic the sounds of their prey, thereby attracting them (55, 56).

The Gray fox, the only canid of the genus *Urocyon*, is medium-sized and omnivorous. It has a total length of 76–112.5 cm, and a whitish fur coat that covers its body. There is also a blackish band on its back and tail (4). The females' smaller size confers a certain sexual dimorphism to the species (57). Due to its excellent food adaptability, it lives throughout the Americas, from southern Canada to northern South America (58), preferentially inhabiting mixed pine forests (57). In North America, Gray foxes live sympatrically with coyotes and are preyed upon by them (59).

Except for the Gray fox, all South American foxes belong to the *Lycalopex* genus (31). The Hoary fox (*L. vetulus*) is present only in Brazil, predominantly in the Cerrado and in transition areas within the Pantanal and Caatinga (60). It has grayish fur on the back of the head and body and a characteristic yellowish belly. Adult males may have a darker dorsomedial band. Due to the similarity of coat, it can be confused with Crab-eating fox and Pampas fox. Therefore, it is essential to consider its more diminutive size for differentiation (61), as it is the smallest neotropical canid (11). With a predominantly insectivorous diet (62), they hunt alone, in pairs, or even in family groups of three to five individuals (11).

The Pampas fox (*L. gymnocercus*) is a medium-sized fox (63, 64) with sexual dimorphism due to males being larger and heavier than females (49). Due to its generalist eating habits, it is well-adapted to various South American biomes in southern Brazil, eastern Argentina, eastern Bolivia, northern Rio Negro Argentina Province, and western Paraguay (65). This species feeds on small and medium-sized rodents, hares, birds, armadillos, skunks, lizards, native fruits, carrion, and garbage (65), has solitary habits, and shelters in burrows during the day (65). The coat on the head and back is reddish with a darker band in the dorsomedial region, extending to the end of the tail, which is relatively long, thick, and gray; in the ventral area, the coat varies from light gray to white. The ears are relatively large, triangular, wide, and reddish (64).

Culpeo (*L. culpaeus*), the largest fox of the genus *Lycalopex* among wild South American canids, is surpassed in size only by the Maned wolf (66). It is distributed from Ecuador to southern Chile, adapting well from desert to forest (4, 67). It has whitish-gray fur on the dorsal area and above the shoulder, whereas on the head, neck, ears, and legs, the tone can be brown or red. The tail, long and thick, has black fur at its tip. It is omnivorous but, unlike other carnivores, defecates in open spaces and does not cover its waste (13).

The Darwin's fox (*L. fulvipes*) has a short and thin snout that, despite its small size, has a robust appearance, elongated body, and short legs with a fur coat that varies from brown to black (68). It is present in humid forest areas of Chile, with only three known groups, located in the Chiloé Island, coastal mountains of Nahuelbuta National Park (26), and Valdivian Coastal Reserve (69), respectively. Darwin's fox is considered an opportunistic omnivore; its diet varies with region and season of the year (68). It is believed that they can form expanded groups under food scarcity and territorial restrictions (13).

The Chilla (*L. griseus*) is a medium-size fox widespread in plains and mountains on both sides of the Andes in Chile and Argentina (Group, Canid Specialist., 2016). It was introduced to Tierra del Fuego in 1951 to control an excessive population of European rabbits (*Oryctolagus cuniculus*) that were causing environmental imbalance (70). Its body, measuring up to 60 cm with a tail up to 36 cm, is covered by yellowish-gray dorsal fur, with sparse white and black hairs. In the ventral region, the fur is whitish reddish-brown. It preferentially inhibits dry and cold climate biomes close to mountain formations such as the Patagonian steppe (7); however, it is very adaptable and can live in hostile habitats such as the Atacama Desert and the cold and rainy forests of Tierra del Fuego (71). The Chilla is omnivorous and has a varied diet of small rodents, arthropods, birds, and fruits (71).

The Sechuran (*L. sechurae*) fox, present from southwest Ecuador to central-west Peru, occupying the Sechura desert (7), is considered omnivorous and can survive prolonged intervals of feed and water restrictions (72). It has a small head, with a short snout and long ears, covered on its rostral face with gray fur, except around the eyes where the tones are reddish-brown. They are solitary animals, rarely seen in groups, except when they form couples during the reproductive season.

Additional characteristics of the South American canids are shown in Tables 1, 2.

**Table 1:** General features of South American canids.

**Species**	**Red list category**	**Chromosome**	**Body weight**	**Dental form**
		**number**	**(kg)**	**I; C; P; M = total**
Crab-eating fox	Least concern	74 (73)	5.7 (4)	3/3; 1/1; 4/4; 2/3 = 44 (35)
Maned wolf	Threatened	76 (74)	25-30 (75)	3/3; 1/1; 4/4; 2/3 = 42 (39)
Hoary fox	Threatened	74 (74)	03.3 (4)	3/3; 1/1; 4/4; 2/3 = 4 (60)
Pampas fox	Least concern	74 (75)	04.4 (4)	3/3; 1/1; 4/4; 2/3 = 42 (75)
Short-eared dog	Threatened	74–76 (76)	09.5 (4)	3/3; 1/1; 4/4; 2/3 = 42 (76)
Bush dog	Threatened	74 (74)	08.0 (2)	3/3; 1/1; 4/ 4;1/2 = 38 (4)
Culpeo	Least concern	74 (77)	09.7 (4)	3/3; 1/1; 4/4; 2/3 = 42 (77)
Darwin's fox	Endangered	–	03.1 (4)	3/3; 1/1; 4/4; 2/3 = 42 (68)
Chilla	Least concern	74 (73)	03.6 (4)	–
Sechuran fox	Threatened	74 (78)	03.6 (4)	3/3; 1/1; 4/4; 2/3 = 42 (79)
Gray fox	Least concern	66 (80)	09.0 (2)	3/3; 1/1; 4/4; 2/3 = 42 (81)
Falkland Island fox	Extinct	66 (80)	–	–

**Table 2:** Biomes occupied by South American canids.

**Scientific name**	**Species**	**Biomes**
Cerdocyon thous	Crab-eating fox	Atlantic Forest, Cerrado, Pantanal, Caatinga, Pampas, and tropical forest (34, 36)
Chrysocyon brachyurus	Maned wolf	Cerrado and transition between Cerrado and Atlantic Forest (38, 41)
Atelocynus microtis	Short-eared dog	Amazon Region (4)
Speothos venaticus	Bush dog	Amazon Region, Cerrado, Atlantic Forest, and Pantanal (51)
Urocyon cinereoargenteus	Gray fox	Savanna (71)
Lycalopex vetulus	Hoary fox	Cerrado and in transition areas between Pantanal and Caatinga (60)
Lycalopex gymnocercus	Pampas fox	Pampas, Steppe, and tropical rainforest (65)
Lycalopex culpaeus	Culpeo	Tundra and Montane Forest (4, 67)
Lycalopex fulvipes	Darwin's fox	Humid forest areas of Chile (26, 69)
Lycalopex griseus	Chilla	Tundra, Montane Forest and steppe (71)
Lycalopex sechurae	Sechuran fox	Tundra and Montane Forest (7)

## Reproductive characteristics

### Reproductive physiology

Little is known about the reproductive physiology of wild South American canids. The scientific literature on the subject is scarce and full of gaps. For example, compared to the information available for domestic dog, almost nothing is known about their puberty and age at sexual maturity, reproductive endocrinology, estrous cycle, sexual behaviors, mating system, or time of ovulation. Information related to the dynamics of the vaginal epithelium throughout the estrous cycle and stages and duration of delivery is also limited. For Darwin's fox and the Short-eared wild dog, even gestation duration is unknown. Available information, generated predominantly from observational studies, is summarized below.

#### Seasonality

Reproductive seasonality is a vital strategy to concentrate births in times of greater food availability and environmentally favorable for offspring survival (82–84). Most wild South American canids are considered seasonally polyestrous, with two reproductive phases: anestrus and breeding seasons. Except for the Maned wolf that is a shot-day-breeder (autumn-winter breeder) (39, 85, 86), most of them, such as the Crab-eating fox (87), Chilla (33), Gray fox (33), Short-eared dog (49), Darwin's fox (68), and Sechuran fox (88), are considered long-day breeders, with a breeding season during late winter and spring. Exceptionally, the Crab-eating fox may give birth twice in the same year, depend on its geographic location and food availability (37). The Hoary fox (89), Pampas fox (90), and Culpeo (52) are monoestrus species, mating during the winter or spring. Of these, the Hoary fox starts its breeding season early, in July (89).

Distinct from the others, the Bush dog is annual polyestrous, although ovarian activity occurs only in the dominant female of the group, being suppressed in subordinate females (84, 91, 92), similar to wolves (81). Unlike other canids, the presence of a male was associated with shortened interestrus intervals and increased numbers of estrous cycles (92).

Little is known about effects of seasonality on male reproduction and sperm production. Based on testosterone data in Bush dogs, it was concluded that sperm production is year-round and not seasonal (84). However, in Maned wolves, blood testosterone concentrations are stable throughout the year, although they have a higher seminal quality index and greater scrotal circumference during the breeding season (2). In Gray foxes, sperm epididymal reserve increases during the reproductive season (93).

During the breeding season, the Maned wolf male increases vocalization and territorial marking with urine (39). The male effect is also described in Bush dogs, where the male presence shortens the interestrus interval (91).

#### Estrous cycle

Information regarding the estrous cycle of the wild canids is scarce and inconsistent. Most of the information is related to the Maned Wolf, in which the follicular phase of estrous cycle seems to be similar to the bitch, with proestrus lasting ~14 days (94), estrus ranges from 1 to 10 days (95), and mating lasting up to 14 min (94, 96). During proestrus, there is vaginal swelling and secretions, as well as increases in social solicitatious behavior (94). In an ultrasonographic survey, the ovaries were ~1.10 cm long and ~0.7 cm wide, although the uterus was not visualized (97). In this species, there is also evidence that ovulation is induced and only occurs in the presence of the male (98), probably triggered by specific volatile organic compounds present in male urine (99), as several volatile organic compounds present in the urine of male Maned wolf are considered semiochemicals (100).

Regarding the Crab-eating fox, it is only known, from an endocrine and colpocytological study, that the estrous cycle phases are like those of the bitch (*Canis lupus familiaris*), except for the absence of bleeding during proestrus (101). Estrus lasts 3 days and, similar to what happens in the dog, during the mating, the penis is retained for 5–8 min when the breeding pair assumes a back-to-back position (37). Plasma progesterone concentration reaches its maximum value (~46 ng/mL) on the 10th gestational day (101).

The long non-gestational diestrus (~75 days), a particular feature of the bitch estrous cycle (77), is only described in Hoary fox (89) and Culpeo females (102).

#### Andrology

Knowledge about andrology of South American canids is minimal, and there are no controlled studies regarding male reproductive endocrinology, puberty, and sexual maturity.

Ultrasonographic descriptions of male reproductive tract are only available for Maned wolf and Crab-eating fox. The ultrasonographic appearance of the testes in Maned wolves is quite different from that of the dog, with a hypoechoic coarse echotexture and mediastinum slightly echogenic and poorly defined. The testes are 2.87 cm long and 1.22 cm wide. Echogenicity of the prostate is similar to that of surrounding tissues, making it difficult to visualize (97). In Crab-eating fox, the prostate is localized caudal to the bladder with a bilobed, regular contour, with homogeneous parenchyma and central prostatic urethra. Spectral Doppler ultrasonography revealed that testicular and capsular arteries had biphasic blood flow with evident systolic peaks, followed by a gradual reduction of diastolic flow (low vascular resistance). In contrast, intratesticular arteries have monophasic blood flow patterns without marked flow during diastole (103).

Semen collection techniques and sperm characterization are the subjects most addressed; however, the surveys and species are limited. Electroejaculation, the method of choice for semen collection in wild carnivores (104), has been adapted to various species (105–107). However, electroejaculation seems to be ineffective in most South American canids, as there is almost always contamination of the ejaculate with urine (108, 109). This represents a critical obstacle for semen characterization and obtaining physiologic sperm samples for artificial insemination or cryopreservation. Urinary contamination of semen is known to cause severe abnormal sperm morphological changes and necrospermia.

There are only a few reports of semen collection by electroejaculation in Maned wolf (110, 111) or urethral catheterization in Maned wolf and crab-eating fox (112, 113). Collection of seminal samples by urethral catheterization of males pre-treated with a medetomidine and ketamine combination seems to be a promising technique for obtaining seminal samples from wild canids *in situ* (112, 113). However, all attempts to collect semen from Maned wolf and Crab-eating fox by electroejaculation or urethral catheterization performed in our laboratory were unsuccessful and/or samples were contaminated with urine (unpublished data). In Crab-eating foxes, attempts at urethral catheterization were monitored by abdominal ultrasonography to ensure that the catheter tip was positioned caudally to the bladder neck, at the *colliculus seminalis* region, using the median portion of the prostate as a reference.

The difficulty of obtaining ejaculates in South American canids has been overcome in Maned wolf (83, 114) and Crab-eating fox (103) using seminal collection by digital stimulation of the penis, similar to what is done in domestic dogs. This approach has already allowed seminal characterization in captive Crab-eating fox (103) and Maned wolf (83), determination of sperm seasonal changes (83), and cryopreservation of seminal samples (114) in Maned wolf. It is worth noting that this technique required prior conditioning of the Crab-eating fox (103) and safety precautions since the animals are neither sedated nor anesthetized. However, in the Maned wolves, there was no necessity for conditioning. The males were physically restrained with a muzzle and catch pole, and seminal samples were obtained in the first attempt (83). Semen characteristics of Crab-eating fox and Maned wolf are summarized in Table 3.

**Table 3:** Mean (± SD) of semen characteristics of South American canids.

**Semen collection method**	**Semen characteristics**	**Species**	
		**Crab-Eating fox**	**Maned wolf**
Eletroejaculation	Volume (mL)	–	2.0 (0.6) (111)
	Total motility (%)	–	59.8 (4.9) (111)
	Sperm concentration (10^6^ × spz/mL)	–	43.4 (18.2) (111)
Pharmacological induced ejaculation	Volume (mL)	0.04 (21.98) (113)	0.1 (112)
	Total motility (%)	40.0 (29.01) (113)	40.0 (112)
	Sperm concentration (10^6^ × spz/mL)	277.6 (298.7) (113)	10.0 (112)
Digital stimulation	Volume (mL)	0.4 (178.4) (103)	1.3 (1.2) (83)
	Total motility (%)	68.0 (6.1) (103)	76.1 (23.9) (83)
	Sperm concentration (10^6^ × spz/mL)	463.7 (594.4) (103)	73.9 (87.2) (83)
Epididymal harvest	Volume (mL)	–	–
	Total motility (%)	–	95.0 and 62.5* (115)
	Sperm concentration (10^6^ × spz/mL)	–	25.4 and 23.5* (115)

Spz, spermatozoids; *Sperm collection during breeding season.

Other techniques employed to study male reproductive physiology, particularly spermatogenesis, sperm production, and testicular disorders, are histological evaluation of testicular parenchyma, preparation of smears, and epididymal sperm counts. These approaches enabled characterization of the testicular morphology of Crab-eating fox (116, 117) and Hoary fox (116, 118) (Table 4), sperm production in the adult Maned wolf (119), and enumeration of epididymal sperm in Gray fox (120).

**Table 4:** Gonadosomatic index and tissue proportion of the testicular tubular and intertubular compartments in *Cerdocyon thous* and *Lycalopex vetulus*.

**Scientific name**	**Species**	***N***	**Gonadosomatic**	**Seminiferous tubules**	
			**Index**	**Intertubular tissue**	**Seminiferous tubules**
			**(%)**	**(%)**	**(%)**
Cerdocyon thous	Crab-eating fox	6	0.07 (0.02) (109)	12.7 (5.3) (109)	87.5 (5.2) (109)
		6	–	15.64 (2.84) (108)	86.96 (2.47) (108)
Lycalopex vetulus	Hoary fox	5	–	13.04 (2.47) (108)	84.37 (2.84) (108)

There are only two reports of seminal cryopreservation in Maned wolf. In the first study, sperm were frozen with an egg yolk extender containing 1 M dimethyl sulfoxide or 1 M glycerol. The use of dimethyl sulfoxide resulted in higher post-thawing motility (20.0 ± 1.9 vs. 13.5 ± 2.1%) and plasma membrane integrity (51.2 ± 1.7 vs. 41.5 ± 2.2%) than glycerol (111). In the other study, conducted by our group, semen was frozen with a TRIS egg yolk extender containing 7% glycerol; post-thawing sperm motility was >55% (114). In other species of South American canids, there are apparently no reports on sperm cryopreservation.

#### Sexual behavior and mating systems

Little is known about wild South American canids' sexual behaviors and mating systems; however, most findings indicate that Pampa fox (121), Chilla (71), Culpeo (122), and Maned wolf (39, 123) have solitary habits and males and females only pair during mating season. Crab-eating fox (124), Maned wolf (39), Pampa fox (63), Hoary fox, and Gray fox (81) are monogamous. However, even though the Gray fox generally has monogamous behavior, polyandry is often apparent (125).

Unlike the above-mentioned species, Bush dog (4) and Chilla (13) are polygamous and form harems; however, reproductive activity is suppressed in subordinate females by the presence of the dominant female of the family group. In the Bush dog, the couple separates from the group at the time of copulation (4).

#### Pregnancy and births

The duration of gestation of wild South American canids and the number of offspring born in each litter are generally similar to those of the bitch, with reported data summarized in Table 5 (4, 6, 7, 10, 11, 33, 39, 51, 86, 126, 127).

**Table 5:** Pregnancy duration and litter size of South American canids.

**Species**	**Duration of pregnancy (days)**	**Litter size**
Crab-eating fox	52–59 (4, 11)	3–6 (6)
Maned wolf	62–66 (4, 39, 77)	1–5 (4, 39)
Hoary fox	50 (10)	2–5 (7)
Pampas fox	55–60 (4)	3–5 (6)
Short-eared dog	–	2–3 (51)
Bush dog	67 (33); 60–83 (4)	1–6 (6)
Culpeo	55–60 (4, 33)	3–8 (6)
Darwin's fox	56 (118)	2–3 (119)
Chilla	53–58 (4)	2–4 (6)
Sechuran fox	–	–
Gray fox	51–63 (4)	1–10 (6)

In general, pregnancies range from 52 to 60 days, being longer in the Maned wolf, which can be up to 65 days (4, 45), and in the Bush dog, with conflicting reports that it can vary from 65 to 83 days (4, 91). Parturition in Short-eared Dog (49), Crab-eating fox (37), Maned wolf (45, 128), Darwin's fox (68), and Chilla (13) occurs in burrows, where the young are kept during initial development. Like domestic dogs, eyes, and auditory canals of Maned wolf puppies only open around the ninth day of life (1, 45, 129).

In Maned wolf, the first parturition can occur as early as at 1.5 years of age, but predominantly happens at 4 years, and pregnancies can be established until 10-12 yr, although uncommon, being more usual from 3 to 8 years (95).

The causes of embryonic and fetal loss and neonatal deaths are unknown in wild canids. There is only one report that Maned wolf females that experience neonatal losses have lower concentrations of progesterone metabolites in their feces during the second half of pregnancy than those whose offspring survived (130).

Behavior monitoring can be used as a non-invasive tool to distinguish mating success in captive Maned wolf. Although the pair's interactive behavior patterns are similar during estrus, approach behavior is only maintained in the post-estrus period when the female becomes pregnant (131).

#### Breastfeeding and parenting

Except for the Sechuran, in which births occur without the presence of the male (72), in other wild South American canids, couples established during the breeding season seem to remain together when pups are suckling. This behavior appears to be significantly favored by monogamy and couple stability (92). In Crab-eating fox, the young remain with their parents, forming extended family groups (124). Similarly, the Maned wolf has stable parental relationships, and males can collaborate during parturition and early development of the young (39, 44). Shared parental care is also reported for the Hoary fox (60), Pampas fox (132), Bush dog (56), Chila (71), and Darwin's fox (122). In Darwin's fox, while the father's parenting care increases, maternal care progressively decreases (68). Male parental behavior in canid appears to be stimulated by a seasonal rise in plasma prolactin concentrations. This phenomenon is well-documented in the Gray wolf, but not yet described for other South American canids (92).

After weaning, which occurs at 4 months of age in Hoary fox (11) and in Darwin's fox (68), 2 months in Pampas fox (4), 2.5 months in the Bush dog (4), and 30–37 days in Coupe (33), family groups remain stable. This arrangement lasts until 6–7 months postpartum in Short-eared dog (50) and Chilla (33), 10 months in Bush dog, when the pups reach sexual maturity (52), and 9–12 months in Crab-eating fox (37). In Short-eared dog, females abandon their offspring and settle in a new foraging area (49). In Hoary fox (61), Bush dog (4), Chilla (76), and Crab-eating fox (124), offspring leave their parents and settle in other areas.

There is no information regarding sexual development of the wild South American canids, except for Gray foxes that reach sexual maturity at 10 months of age but do not always become pregnant in the first year (13).

#### Current situation and perspectives

Physiological knowledge is essential for developing reproductive biotechnologies for establishing germplasm banks for present and future use. However, a broad understanding of the challenges is indispensable to succeed in this task.

The initial objective of this review was to compile the available knowledge regarding reproductive biology of wild South American canids, assess potential use of this information for development of animal conservation programs, and highlight the main obstacles to realization of these proposals. However, it is clear that this possibility remains remote.

The considerable current gaps in the knowledge of reproductive biology of South American canids and their great diversity are substantial obstacles to developing consistent conservation programs. This situation is aggravated by difficult access to knowledge already produced, as some of it was recorded only in local yearbooks of the zoos or zoo-botanical parks.

The current knowledge predominantly derives from observational and descriptive studies regarding social dynamics (133), ecology (89, 134, 135), diet (17, 34, 67), sharing of foraging areas by sympatric canids (38), diet, habitat use, and home ranges of sympatric canids (38, 59, 65, 136, 137), reproductive season (83, 84, 138), parental care (37, 44, 45, 132), time of birth (92, 127), and ontogeny (12, 37). However, basic female reproductive processes such as endocrine patterns, details regarding the estrous cycle, ovulatory mechanisms, physiology of pregnancy, and causes of infertility are still not understood. Concerning andrology, this scenario is even worse. It was disconcerting to realize, for example, that there are no basic studies about male reproductive anatomy and physiology, except for Maned wolf (83) and Crab-eating fox (103).

There are several reasons for the reduced number of studies on reproductive biology of wild South American canids. Unlike domestic animals, in wild animals, simple procedures such as physical examination, blood collection, and ultrasonographic examination are complex due to the need for pharmacological restraint. This creates potential risks for accidents for the animals and people involved in these procedures. Besides this, restraint procedures invariably trigger endocrine and behavioral stress responses (139) that invalidate many physiological data. One approach to overcome these difficulties is using non-invasive methods of monitoring endocrine function by measuring fecal concentrations of sex steroid metabolites. This method was used to determine endocrine and reproductive cycles in Hoary fox (88), Maned wolf (94) and Bush dog (91), and to study fetal losses in Maned wolf (130). However, this assessment is also difficult as it requires monitoring the animals to verify the sample's origin and rapid retrieval to avoid degradation of sex hormones metabolites by fecal microbiota (140–142). These issues are particularly limiting in free-range and nocturnal animals.

Society does not clearly understand the role of wildlife conservation in planetary balance and global One Health. Our current urban life seems to keep us away from this reality and strongly influences private and public policies. Limited public and private investments in research are blamed for the limited knowledge regarding reproduction in the wild South American canids. The appeal to raise funds for studies involving livestock and companion animals is much more substantial, as they are directly related to food production and human wellbeing.

Difficulties in developing efficient conservation plans go well-beyond just financial issues. These initiatives also depend on specialized labor and are affected by difficulties in priorizing species (143, 144). Wildlife conservation programs require an interdisciplinary team to understand and mitigate the challenges of wildlife sanitary conditions, nutritional requirements, behavioral issues, arrested sexual development, infertility, and low reproductive efficiency (14, 145, 146).

The future of the South American wild canids depends on developing several areas of knowledge. A promising area of study involves development of hormonal protocols for induction of ovarian activity and ovulation. However, on this subject, there is only one report of the use of deslorelin implants (GnRH agonist) for 7–11 days to induce ovarian activity and ovulation in paired Maned wolf females and of the use of recombinant equine luteinizing hormone for ovulation induction in isolated ones (96). Another promising area is the use of knowledge on the reproductive physiology of the bitch (141, 147) to monitor reproductive activity in female wild canids. This approach was used in Maned Wolf females in which the plasma concentrations of anti-Mullerian hormone reflected ovarian activity, being higher in adulthood and during the breeding season (147).

## Final considerations

Current knowledge gaps make it impossible to establish consistent conservative programs and develop reproductive biotechnologies for South American Canidis. This reality is worrying and emphasizes the need to alert the scientific community, society, and governments of the urgent requirement for investments and the potential consequences of failing to act promptly, as most of these animals are currently either vulnerable or threatened. Furthermore, in the broader context, preservation of South American biomes is imperative, as it does not make sense to develop reproductive knowledge and reproductive technologies only for captive specimens. Preserving the environmental conditions, coupled with broader knowledge of reproductive function, are critical to increase the genetic variability, number of free-ranging animals, and ultimately promote planetary One Health.

## Author contributions

JC and JF conceived of the proposed idea and wrote the manuscript. FS, JK, and JF revised and edited the manuscript. All authors contributed to the article and approved the submitted version.

## Funding

Financial support from FAPESP and CAPES (Grant#001).

## Conflict of interest

The authors declare that the research was conducted in the absence of any commercial or financial relationships that could be construed as a potential conflict of interest.

## Publisher's note

All claims expressed in this article are solely those of the authors and do not necessarily represent those of their affiliated organizations, or those of the publisher, the editors and the reviewers. Any product that may be evaluated in this article, or claim that may be made by its manufacturer, is not guaranteed or endorsed by the publisher.
